# Winning and Losing Tree Species of Reassembly in Minnesota’s Mixed and Broadleaf Forests

**DOI:** 10.1371/journal.pone.0061709

**Published:** 2013-04-15

**Authors:** Brice B. Hanberry, Brian J. Palik, Hong S. He

**Affiliations:** 1 Department of Forestry, University of Missouri, Columbia, Missouri, United States of America; 2 USDA Forest Service, Northern Research Station, Grand Rapids, Minnesota, United States of America; University of Zurich, Switzerland

## Abstract

We examined reassembly of winning and losing tree species, species traits including shade and fire tolerance, and associated disturbance filters and forest ecosystem types due to rapid forest change in the Great Lakes region since 1850. We identified winning and losing species by changes in composition, distribution, and site factors between historical and current surveys in Minnesota’s mixed and broadleaf forests. In the Laurentian Mixed Forest, shade-intolerant aspen replaced shade-intolerant tamarack as the most dominant tree species. Fire-tolerant white pine and jack pine decreased, whereas shade-tolerant ashes, maples, and white cedar increased. In the Eastern Broadleaf Forest, fire-tolerant white oaks and red oaks decreased, while shade-tolerant ashes, American basswood, and maples increased. Tamarack, pines, and oaks have become restricted to sites with either wetter or sandier and drier soils due to increases in aspen and shade-tolerant, fire-sensitive species on mesic sites. The proportion of shade-tolerant species increased in both regions, but selective harvest reduced the applicability of functional groups alone to specify winners and losers. Harvest and existing forestry practices supported aspen dominance in mixed forests, although without aspen forestry and with fire suppression, mixed forests will transition to a greater composition of shade-tolerant species, converging to forests similar to broadleaf forests. A functional group framework provided a perspective of winning and losing species and traits, selective filters, and forest ecosystems that can be generalized to other regions, regardless of species identity.

## Introduction

McKinney and Lockwood [1] proposed that anthropogenic disturbance and introduction of exotic species will lead to expansion in abundance and range by winning species and reduction in abundance and range by losing species. Further, they suggested that replacement of a diversity of losing species by a few generalist winning species will be amplified if losers share functional traits, often clustered to a common taxonomy. Plant functional groups are classified based on functional traits of species in response to the environment. Functional groups predict effects of species on ecological function and reflect ecological constraints on plant assembly in communities [2]. Disturbance is one of the central filters of success or failure of plant life history strategies, although different types and attributes of disturbance will favor alternative traits. Moreover, just as the functional group of the most dominant species may determine function of the ecosystem type [3], attributes of the most dominant species may determine the ecosystem type. Therefore, changes in disturbance regimes create the potential for reassembly from an ecosystem type dominated by species that share a set of functional traits to a different ecosystem type dominated by species of another functional group.

A wave of intensive harvest and clearing for agriculture removed most of the overstory in eastern broadleaf and mixed forests, which are transitional between broadleaf and boreal forests, of the United States by 1920, creating region-wide compositional conversion [4]. Harvesters selected economically valuable species and canopy trees, often conifers. Conifers historically were dominant in mixed forests of Great Lakes states, and in part were able to reinforce their position through a positive feedback of forest floor needles, which suppressed available nutrients, making conditions more stressful for nutrient-demanding hardwoods [5]. Harvest of overstory conifers removed seed sources, while intense slash fires killed cones and advance regeneration and burned pine leaf litter [5]–[6]. Light-seeded and sprouting species and species with less timber utility at the time were able to establish in vacant growing space, converting overstory species from conifers. Conversely, in eastern broadleaf forests, canopy removal of oak forests allowed oak advance regeneration in the understory to recruit into the canopy [7]. However, after subsequent competitive failure of oaks [8], harvest has accelerated turn-over of oaks to faster growing species [9].

Effective fire suppression began about 1920 in the United States and since then, fire-sensitive species have been replacing oak and pines, perhaps rendering open oak and pine forest ecosystems artifacts of discontinued fire regimes [10]. Open forest ecosystems, generally of pine or oak in temperate regions, are stabilized by a fire regime irregular enough to allow young trees with fire-tolerant traits to occasionally escape fire mortality but regular enough to kill fire-sensitive tree species [4], [11]. In contrast, dense, multilayered forests develop too much biomass to be fire-dependent [11]. Fire suppression allowed fire-sensitive species, which allocate resources to above-ground shoot growth, to out-compete oak and pine species, which allocate resources to below-ground root growth [4]. Without regular fire, fire-sensitive species are able to progressively engineer mesic and fire-resistant conditions, by increasing tree density and shadiness and thereby retaining moisture while reducing wind, light, and light-demanding herbaceous species (i.e., fine fuels) within the site, facilitating recruitment of species with greater shade tolerance [4].

**Figure 1:**
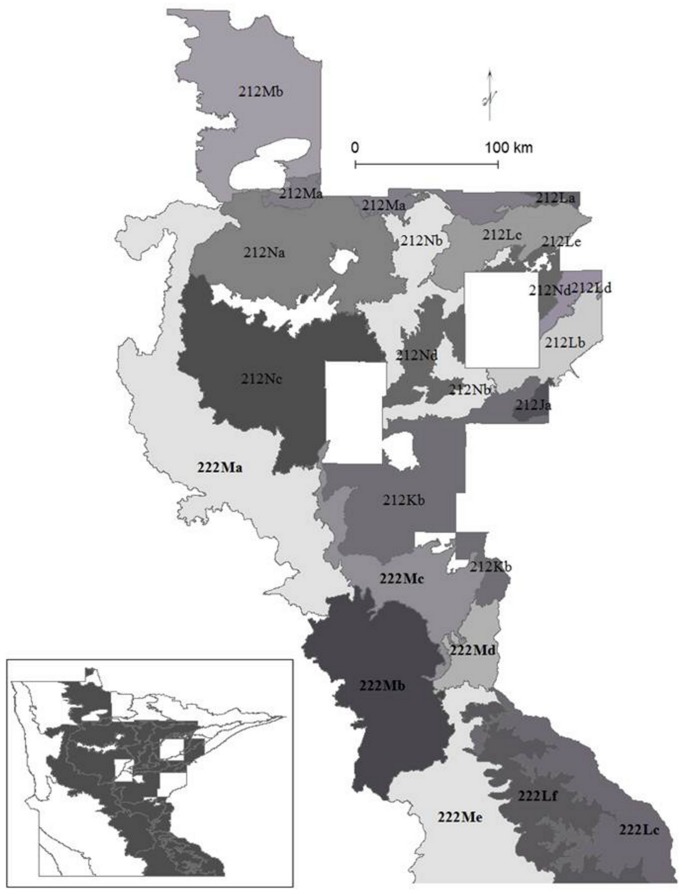
Ecological subsections (shaded) of the Eastern Broadleaf Forest (222 prefix) and the Laurentian Mixed Forest (212 prefix) of Minnesota.

Given these disturbances and consequent reassembly of ecosystems, our first objective was to quantify the extent and trajectory of reassembly of forest ecosystems, including identification of winning and losing tree species, using historical and current forest surveys in the two forested provinces of Minnesota, the Eastern Broadleaf Forest and the Laurentian Mixed Forest (Figure 1). The spatial and temporal frame of this study allowed us to determine changes in composition, distributions, and environmental site factors, which indicate expansion or contraction of species in abundance, range, and environmental gradients.

We then examined common functional traits of winning and losing species in the context of changing disturbance filters. We expected that functional groups driven by harvest will have a greater proportion and distribution of early-successional fast-growing species, as measured by reduced shade tolerance rather than dispersal, than historical forests. Although harvest does not directly remove understory regeneration, harvest is selective; therefore, we also expected to see shifts among early-successional species and loss of conifers. We expected that for fire suppression to be a primary driver of functional groups, dominant fire-dependent oaks and pines will decrease in composition and distribution and fire-sensitive species will increase in composition and distribution. Furthermore, proportion of species with greater shade tolerance should increase due to release from fire disturbance that historically removed trees and reversed transitional stages.

## Methods

### Tree Surveys

The General Land Office (GLO) instituted the Public Land Survey System of townships and ranges in 1812 to survey, map, and sell unsettled territories following a systematic method [12]. The GLO divided townships into 36 sections of 1.6×1.6 km area. Surveyors recorded species, distance, bearing, and diameter for two to four bearing trees at the corners and middle of each section line (i.e., every 0.8 km). The GLO surveys contained about 340,000 trees, surveyed between 1847 to 1908, within the two forested provinces of Minnesota from the GLO dataset (J. Almendinger, Minnesota Department of Natural Resources, http://deli.dnr.state.mn.us). We used Public Land Survey corners and tree distance and bearing to locate the trees. About 90,283 trees had no azimuth, and of these, we kept 33,547 trees within a distance less than 5.7 m and gave the trees a neutral bearing of 45 degrees. By our calculations, this produced a maximum offset distance of 4 m.

There are potential sources of error in GLO surveys. Surveyors selected tree species at survey points and thus, historical forest composition may have differed from selected tree surveys. However, surveyors also were instructed to record tree species encountered along the section lines, although line data are not publicly available for Minnesota. Line surveys supply an alternative sample of historical composition. When comparing percent composition between trees encountered along section line and trees selected at survey points, mean absolute difference was 0.8% and maximum difference was 3.2% for a common tree species in the Missouri Ozarks [13]. These values provide a range of variation that can occur with different sampling methods. Surveyors also may have mis-identified tree species or identified trees to genera. These biases contribute to the value of functional groups rather than reliance on imperfect species counts. A source of error for species distribution models may arise from locational accuracy, because the location of Public Land Survey corners may have drifted over time and in Minnesota, azimuth information for some species was not available. However, measurements to some extent always contain error and large sample sizes should correct for trees that are located in unusual conditions.

The U.S. Forest Service Forest Inventory and Analysis (FIA) surveys fixed plots, visiting 20% of plots every year in a five year cycle. We used the latest complete cycle from 2004–2008. There is about one plot per 1200 hectares in the forested provinces of Minnesota. Available FIA plot locations (FIA DataMart, www.fia.fs.fed.us/tools-data) are perturbed (i.e., location moved) to protect landowners. However for use in modeling, the USDA Forest Service joined environmental variables to plots.

We grouped trees into categories due to categories present in the historical surveys (Table 1). Most of the trees we did not use were unidentified pines and oaks, as well as understory species such as ironwood (*Ostrya virginiana*). We selected live trees that were ≥7.6 cm in diameter, the smallest diameter in GLO surveys, to make the diameter distributions more comparable.

**Table 1:** Tree species/group composition for GLO (1847–1908) and FIA (2004–2008, ≥7.6 cm DBH) surveys in the Laurentian Mixed Forest (LMF) and Eastern Broadleaf Forest (EBF) of Minnesota.

		LMF				EBF				
		GLO		FIA		GLO		FIA		Shade
Species or group		Count	%	Count	%	Count	%	Count	%	tolerance^1^
American basswood	*Tilia americana*	2326	1.00	3062	2.75	4359	5.21	1265	10.85	3.98±0.15
ashes	*Fraxinus nigra, F. pennsylvanica,* *F. americana*	4307	1.85	9646	8.65	2795	3.34	995	8.53	2.46±0.21–3.11±0.11
aspens	*Populus tremuloides, P. balsamifera,* *P. grandidentata*	29384	12.64	25441	22.83	11703	13.98	1568	13.44	1.21±0.27 −1.27±0.14
balsam fir	*Abies balsamea*	13599	5.85	8872	7.96	NA	NA	NA	NA	5.01±0.09
birch	*Betula papyrifera*	25251	10.86	8965	8.04	1449	1.73	332	2.85	1.54±0.16
boxelder	*Acer negundo*	NA	NA	NA	NA	50	0.06	861	7.38	3.47±0.1
cherries	*Prunus pensylvanica, Prunus serotina*	NA	NA	NA	NA	217	0.26	228	1.95	2.46±0.34
eastern white pine	*Pinus strobus*	13586	5.84	957	0.86	237	0.28	114	0.98	3.21±0.2
elms	*Ulmus americana, U. rubra, U. thomasii*	3553	1.53	878	0.79	8372	10.00	1474	12.64	3.14±0.12 −3.31±0.19
hickories	*Carya cordiformis, C. ovata*	NA	NA	NA	NA	645	0.77	248	2.13	2.07±0.07 −3.4±0.29
jack pine	*Pinus banksiana*	17535	7.54	3265	2.93	NA	NA	NA	NA	1.36±0.33
maples	*Acer rubrum, A. saccharum,* *A. saccharinum*	6584	2.83	8920	8.00	4901	5.86	1159	9.94	3.44
northern white cedar	*Thuja occidentalis*	11290	4.85	9463	8.49	NA	NA	NA	NA	3.45±0.4
red oaks	*Quercus nigra, Q. ellipsoidalis, Q. rubra*	2431	1.05	2254	2.02	12275	14.67	1049	8.99	2.24±0.76 −2.75±0.18
red pine	*Pinus resinosa*	11299	4.86	4711	4.23	92	0.11	477	4.09	1.89±0.21
spruces	*Picea mariana, P. glauca*	33303	14.32	14698	13.19	191	0.23	34	0.29	4.08±0.18 −4.15±0.17
tamarack	*Larix laricina*	53889	23.17	7209	6.47	3963	4.74	113	0.97	0.98±0.09
walnuts	*Juglans cinerea, J. nigra*	NA	NA	NA	NA	366	0.44	152	1.30	1.88±0.21 −1.93±0.25
white oaks	*Quercus alba, Q. macrocarpa*	3005	1.29	2841	2.55	32070	38.32	1594	13.67	2.71±0.27 −2.85±0.17
yellow birch	*Betula alleghaniensis*	1206	0.52	269	0.24	NA	NA	NA	NA	3.17±0.16

1 Niinemets, Ü., and Valladares, F. 2006. Tolerance to shade, drought, and waterlogging of temperate northern hemisphere trees and shrubs. Ecological Monographs 76∶ 521–547.

### Composition

We determined percent composition of the species groups by ecological province and subsection [14] as a rough measure of relative abundance. There were about 111,000 trees in FIA surveys and 233,000 trees in GLO surveys for the Laurentian Mixed Forest and about 12,000 trees in FIA surveys and 84,000 trees in GLO surveys for the Eastern Broadleaf Forest. We compared only the selected tree species for each province; however by ecological subsection, we compared all species ≥10% in either the GLO or FIA surveys. Although there are trends when comparing percent composition, the trends have a few caveats. The GLO surveys were not random selection of species, so the exact percentage composition is unknown. Furthermore, about 2 to 5 percent of total trees were unidentified oaks and pines, thus oak and pine contribution to composition is underrepresented. Also, due to urbanization, there are few present day trees for comparison in some of the Eastern Broadleaf Forest subsections. As our threshold for determining important trends, a historically common tree species that changes by a three or more composition percentage units (e.g., from 10% to 7% composition) may indicate a change in frequency and a historically rare species that at least triples in composition percentage (e.g., from 1% to 3% composition) may indicate increasing frequency.

### Species Distribution Modeling

We use species distribution models to examine changes in distribution of ranges and site factors for evidence of expansion into new environmental gradients or contraction away from environmental gradients. We excluded species with less than 100 individual trees (before intersection with environmental variables, which further reduced sample size) for distribution modeling. Ultimately, for modeling and prediction, in the Laurentian Mixed Forest province, there were about 93,000 FIA trees and 118,000 GLO trees for 16 species/groups, and in the Eastern Broadleaf Forest province, there were about 13,000 FIA trees and 41,000 GLO trees for 14 species/groups.

We used Soil Survey Geographic (SSURGO) Database (Natural Resources Conservation Service, http://soildatamart.nrcs.usda.gov) polygons as our spatial unit. After processing, there were about 1,000,000 polygons, divided into about 310,000 polygons for the Laurentian Mixed Forest (4,895,238 ha total, polygon mean area of 16 ha) and 750,000 polygons for the Eastern Broadleaf Forest (4,272,893 ha, polygon mean area of 6 ha). Because discontinuous soil polygons share characteristics, our prediction unit was a unique zone of map unit for each county (i.e., soil polygons with similar characteristics), land type association, and geology (Laurentian Mixed Forest mean area was 210 ha, Eastern Broadleaf Forest mean area was 146 ha). Due to lack of soils surveys in seven counties in the Laurentian Mixed Forest, five subsections had reduced areas with environmental information, and we grouped these with the nearest subsections for modeling (Figure 1).

We prepared 16 environmental variables. Seven environmental variables were from the SSURGO tables by map unit for each county (i.e., polygons with similar soil characteristics in a county). Categorical variables were drainage class (very poorly drained to excessively drained) and hydric soil presence. Continuous variables were water holding capacity (cm/cm), pH, organic matter (%), clay (%), and sand (%). From a 30 m DEM (digital elevation model), we calculated seven variables: elevation (m), slope (%), transformed aspect (1+sin(aspect/180π+0.79); [15]), solar radiation (0700 to1900 in 4 hour intervals on summer solstice for re-sampled 60 m DEM), topographic roughness ([16]), wetness index (i.e., where water flows; (ln(flow accum+1)/(tan(((slope deg)3.141593)/180))), and topographic position index (T. Dilts, http://arcscripts.esri.com). We also joined geology and ecological subsection to each individual polygon. Subsections are spatially continuous ecological divisions of the regions developed by experts based on similar climate, soils, topography, hydrology, geology, vegetation, and ultimately, fire regime. Although there is some overlap with other variables, in that soils and topography should vary by subsection, the large scale, spatial continuity, and unique nature of ecological subsections separate this variable from finer resolution, scattered, and repeatable soil and topographic variables.

We (i.e., the USDA Forest Service) joined all survey points or plots to the soil survey polygons. We randomly selected 0.67 of the joined polygons with each species, up to 2500 polygons, for modeling, and held back the rest for prediction and validation. We randomly selected up to 2500 polygons without a recorded species presence for modeling from the modeling sample.

We applied random forests classification ([17]–[18]), a classification method based on bootstrap aggregation (bagging) by the majority of classification trees, which were modeled using random samples of both predictor variables and training data. We used the randomForest package [19], in R statistical software [20], with the sampsize option (which is sampled without replacement*),* where we set the bag fraction, or subsampling rate, at 0.67 of the selected polygons with the species. We then specified 0.25 of that value of the selected polygons with unknown presence or absence of the tree species. We set the number of trees at 1000 and the number of variables randomly sampled at each split as the square root of the number of predictors.

We assessed model accuracy by measuring the proportion of predicted presences that were correctly identified (i.e., the true positive rate; ROCR package [21] in R). We compared predicted probabilities between the same species for GLO and FIA models using Pearson’s correlation coefficient (SAS software, version 9.1, Cary, North Carolina; Proc Corr).

We assessed environmental variable importance, ranked by random forests. We re-scaled variable importance values, by assigning the top value as 1 and divided other values by the original value of the top variable. We focused on similarities between the most influential five variables of the 16 variables. For the most influential two variables from GLO and FIA surveys, we averaged the variable values for predicted probabilities ≥75% and compared these values to means of variable values for predicted probabilities <75%. After grouping the predictions into 4 bins (0–25%, 25–50%, 50–75%, 75–100%), we mapped the distributions (please contact the authors for maps or GIS layers). We looked for general patterns in changes over time.

### Functional Traits

We determined the composition of gymnosperm species compared to angiosperm species in the Laurentian Mixed Forest only and fire-dependent oak and pines compared to fire-sensitive species for trees of diameter ≥12.7 cm in historical forests and current forests, and potentially the trajectory of future forests using diameter <12.7 cm. We applied Niinemets and Valladares’ [22] measurements of shade- tolerance on a scale of increasing tolerance from 1 to 5 and found mean values for trees of diameter ≥12.7 cm in historical forests and current forests, and the trajectory of future forests using diameter <12.7 cm.

## Results

### Compositional Change

In the Laurentian Mixed Forest, tamarack decreased from the most abundant trees species to a minor component (from about 23% to 6% of composition), while aspens increased to the most dominant tree species group (from about 13% to 23% of composition; Table 1; in FIA surveys, quaking aspen comprises about 85% of the aspens). White pine decreased from 6% to 1% of composition and jack pine decreased from 8% to 3% of composition. Ashes (primarily black ash), maples, and white cedar increased by about 4% to 7% in overall composition (e.g., ashes increased from 2% to 9% of composition). Aspens increased and tamarack decreased in all Laurentian Mixed Forest subsections, and although tamarack, spruces, birch, and jack pine were the most common tree species by subsection historically, aspens currently are the most common tree species group in eight of twelve subsections (Table 2).

**Table 2:** Composition percentage of GLO and FIA trees (≥10% for at least one survey, trees ≥7.6 cm DBH) by ecological subsection in the Laurentian Mixed Forest.

	GLO		FIA	
Species	Count	%	Count	%
212 Kb\Mille Lacs Uplands				
tamarack	7139	20.29	373	3.11
aspens	4143	11.77	2941	24.49
birches	3774	10.73	786	6.54
maples	2910	8.27	2235	18.61
ashes	1568	4.46	1583	13.18
212 La\Border Lakes				
spruces	5281	18.94	2127	17.10
birches	4345	15.58	1720	13.83
jack pine	4021	14.42	982	7.89
aspens	3909	14.02	2703	21.73
balsam fir	2503	8.98	1452	11.67
212 Lb\North Shore Highlands				
birches	3191	18.96	1484	14.47
spruces	2969	17.64	1339	13.06
balsam fir	2552	15.16	1345	13.12
tamarack	1970	11.70	199	1.94
white pine	1723	10.24	47	0.46
white cedar	1627	9.67	702	6.85
aspens	995	5.91	2049	19.98
maples	563	3.35	1591	15.52
212 Lc\Nasheswauk Uplands				
birches	1683	17.50	404	9.04
spruces	1624	16.89	506	11.32
tamarack	1310	13.62	223	4.99
balsam fir	799	8.31	565	12.64
aspens	797	8.29	1242	27.78
212 Ld\Toimi Uplands				
spruces	1083	26.75	741	29.62
birches	865	21.37	293	11.71
tamarack	732	18.08	64	2.56
white pine	419	10.35	8	0.32
balsam fir	266	6.57	377	15.07
aspens	207	5.11	432	17.27
212 Le\Laurentian Uplands				
spruces	1935	30.11	1585	34.03
birches	956	14.87	617	13.25
jack pine	955	14.86	340	7.30
tamarack	832	12.95	173	3.71
balsam fir	599	9.32	526	11.29
aspens	408	6.35	565	12.13
212 Ma\Littlefork-Vermillion Uplands				
spruces	5077	25.31	2290	20.93
tamarack	4187	20.87	473	4.32
aspens	3216	16.03	2554	23.34
white cedar	1818	9.06	1971	18.01
ashes	423	2.11	1327	12.13
212 Mb\Agassiz Lowlands				
tamarack	17062	52.14	2929	19.41
spruces	6254	19.11	2718	18.02
aspens	3443	10.52	2722	18.04
white cedar	2029	6.20	3105	20.58
212 Na\Chippewa Plains				
tamarack	4539	19.59	775	6.75
aspens	3461	14.93	3073	26.78
jack pine	2832	12.22	254	2.21
212 Nb\St. Louis Moraines				
tamarack	3098	17.41	398	4.04
birches	2954	16.60	735	7.47
aspens	2000	11.24	2343	23.81
spruces	1839	10.33	739	7.51
maples	1178	6.62	1330	13.51
ashes	350	1.97	1051	10.68
212 Nc\Pine Moraines & Outwash Plains				
jack pine	6023	19.73	799	7.01
aspens	5191	17.00	3188	27.97
red pine	4270	13.98	931	8.17
tamarack	3837	12.57	357	3.13
212 Nd\Tamarack Lowlands				
tamarack	6489	39.85	1103	16.80
spruces	2780	17.07	1364	20.77
birches	1605	9.86	266	4.05
aspens	1525	9.36	1249	19.02
ashes	249	1.53	809	12.32

For the Eastern Broadleaf Forest overall, white oak decreased from the most dominant tree to a moderate component (from about 38% to 14%) while ashes, American basswood, and maples increased their relative abundance, each gaining about 5% in overall composition (Table 1). Two species that were extremely rare (less than 100 records) in historical surveys, red pine and boxelder, increased to 4% and 7% composition respectively in the FIA surveys. Red oaks and tamarack decreased by at least 4% in overall composition. Although historically rare, cherries, hickories, walnuts, and white pine may have at least tripled in composition (e.g., cherries increased from 0.3% to 2%). By subsection, and with few FIA trees to compare, white oak composition decreased from as great as 80% of historical composition to 10% of current composition (Table 3). Boxelder, an extremely rare tree in historical surveys, became a moderate component (12 to 15%) in three subsections of current forests.

**Table 3:** Composition percentage of GLO and FIA trees (≥10% for at least one survey, trees ≥7.6 cm DBH) by subsection in the Eastern Broadleaf Forest.

	GLO		FIA	
Species	Count	%	Count	%
222 Lc\The Blufflands				
white oaks	8001	64.59	321	10.61
red oaks	2603	21.01	326	10.78
elms	350	2.83	599	19.80
222 Lf\Rochester Plateau				
white oaks	5591	77.29	90	10.38
red oaks	991	13.70	43	4.96
elms	95	1.31	180	20.76
basswood	68	0.94	94	10.84
boxelder	3	0.04	134	15.46
222 Ma\Hardwood Hills				
aspens	7614	24.34	1078	21.33
unknown oaks	5073	16.22	NA	NA
white oaks	3518	11.25	832	16.46
maples	2407	7.69	562	11.12
basswood	1521	4.86	705	13.95
ashes	1252	4.00	561	11.10
222 Mb\Big Woods				
elms	4653	21.18	224	16.40
white oaks	4176	19.01	62	4.54
aspens	2532	11.53	36	2.64
basswood	2468	11.24	140	10.25
red oaks	2264	10.31	81	5.93
maples	2138	9.73	139	10.18
ashes	994	4.53	144	10.54
boxelder	23	0.10	184	13.47
222 Mc\Anoka Sand Plain				
white oaks	3495	37.92	211	11.83
red oaks	3445	37.38	278	15.58
tamarack	880	9.55	18	1.01
aspens	491	5.33	221	12.39
red pine	22	0.24	305	17.10
222 Md\St. Paul-Baldwin Plains				
white oaks	2127	58.85	51	9.62
red oaks	921	25.48	64	12.08
maples	23	0.64	51	9.62
red pine	0	0.00	76	14.34
222 Me\Oak Savanna				
white oaks	5162	79.43	27	9.89
red oaks	671	10.32	19	6.96
maples	23	0.35	68	24.91
boxelder	4	0.06	32	11.72

### Species Distribution Models – Predicted Probabilities and True Prediction Rates

Predicted probabilities for GLO and FIA tree species groups varied. For the Laurentian Mixed Forest, correlation between probabilities of species distributions from GLO and FIA surveys was moderate (mean = 0.60), with a range from 0.85 for red pine to 0.19 for white oaks. For the Eastern Broadleaf Forest, differences were greater. Correlation between the probabilities was low (mean = 0.39), with a range from 0.71 for hickories to −0.08 for cherries. True positive rates for modeling were accurate, particularly for FIA surveys. For the Laurentian Mixed Forest, true positive rates at a 75% threshold ranged from 0.72 to 0.87 (mean = 0.80) for GLO tree species groups and 0.81 to 0.97 (mean = 0.91) for FIA tree species groups. For Eastern Broadleaf Forest, true positive rates at a 75% threshold ranged from 0.71 to 0.97 (mean = 0.82) for GLO tree species groups and 0.67 to 1 (mean = 0.90) for FIA tree species groups. The 0.67 true positive rate was for tamarack, which is currently rare and thus, there were only 28 individuals after intersection with our spatial units.

### Species Distribution Models –influence of Environmental Variables

Important environmental variables generally remained similar for species between GLO and FIA surveys in both provinces, but values for those variables became relatively less important and changed for some species. In the Laurentian Mixed Forest, ecological subsection was the most influential variable and the difference between wetlands (wet and organic) and sandy soils was more important than topography (Table 4). For GLO surveys, subsection was the most common influential variable (one of the five most influential variables for 14 models, overall mean rank of 2.94), followed by geology and water capacity (12 models), wetness index and % sand (7 models), slope and % clay (6 models). For FIA surveys, slope and subsection were one of the five most influential variables in 11 models (subsection had a mean rank of 4.38), followed by water capacity, % sand, and wetness index (10 models each), and % organic matter (8 models).

**Table 4:** Two most influential predictor variables for GLO and FIA species distribution models by species and values of the predictor variables for predicted presence (probabilities ≥75%) and predicted absence (probabilities <75%) of the species in the Laurentian Mixed Forest.

		GLO importance	FIA importance	GLO				FIA			
				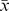	SD	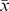	SD	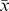	SD	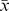	SD
				probabilities ≥75%		probabilities <75%		probabilities ≥75%		probabilities <75%	
American basswood	subsection	1.00									
	geology	0.94									
	aspect		1.00	1.03	0.12	1.14	0.16	1.00	0.09	1.13	0.16
	clay (%)		0.96	13.74	7.20	10.90	10.76	14.44	7.17	11.14	10.23
ashes	geology	1.00									
	elevation (m)	0.86	0.97	375.62	42.62	410.48	46.63	378.44	46.56	405.81	46.01
	sand (%)		1.00	48.54	22.64	59.61	29.82	43.95	22.39	61.67	28.15
aspens	subsection	1.00									
	water capacity (cm/cm)	1.00	1.00	0.16	0.09	0.18	0.12	0.14	0.06	0.22	0.14
	wetness		0.87	5.52	0.84	5.74	0.99	5.40	0.89	5.95	0.89
balsam fir	subsection	1.00	1.00								
	wetness	0.76		5.59	0.84	5.66	0.97	5.58	0.85	5.66	0.95
	pH		0.58	6.26	0.64	6.44	0.59	6.22	0.62	6.43	0.60
birch	wetness	1.00	1.00	5.43	0.81	5.78	0.97	5.15	0.91	5.88	0.84
	subsection	0.99									
	slope		0.89	2.53	1.84	2.43	2.23	3.51	2.55	1.96	1.59
eastern white pine	wetness	1.00	1.00	5.34	0.82	5.83	0.95	4.78	0.62	5.75	0.91
	geology	0.77									
	sand (%)		0.92	54.81	25.15	54.66	28.79	71.34	24.76	52.61	27.04
elms	water capacity (cm/cm)	1.00		0.17	0.08	0.17	0.13	0.18	0.09	0.17	0.13
	subsection	0.99	1.00								
	clay (%)		0.52	14.00	8.15	10.58	10.32	13.59	7.39	10.75	10.93
jack pine	sand (%)	1.00	0.97	73.33	25.55	49.89	25.80	82.79	20.50	51.35	26.31
	geology	0.77									
	water capacity (cm/cm)		1.00	0.12	0.10	0.18	0.11	0.10	0.08	0.18	0.11
maples	wetness	1.00	1.00	5.26	0.78	5.89	0.94	5.12	0.83	5.92	0.85
	subsection	0.93									
	slope		0.78	2.76	1.98	2.28	2.13	3.25	2.44	2.04	1.72
northern white cedar	subsection	1.00									
	aspect	0.62	0.68	1.16	0.17	1.07	0.14	1.24	0.15	1.09	0.15
	organic matter (%)		1.00	20.55	28.52	9.16	19.74	41.16	31.09	9.97	20.61
red oaks	subsection	1.00	1.00								
	wetness	0.53	0.67	5.07	0.72	5.96	0.88	4.98	0.77	5.92	0.85
red pine	subsection	1.00									
	elevation (m)	0.87		426.96	30.31	382.09	47.98	420.64	28.37	392.57	48.93
	sand (%)		1.00	62.84	29.11	51.42	26.02	82.69	17.35	51.98	26.71
	water capacity (cm/cm)		0.77	0.16	0.12	0.18	0.11	0.09	0.06	0.18	0.11
spruces	subsection	1.00									
	water capacity (cm/cm)	0.76	0.93	0.22	0.14	0.15	0.09	0.30	0.16	0.15	0.09
	organic matter (%)		1.00	21.90	29.52	7.93	17.83	41.41	33.82	8.26	17.78
tamarack	wetness	1.00		6.30	0.74	5.33	0.84	6.55	0.59	5.53	0.90
	organic matter (%)	0.81	1.00	26.45	29.85	5.16	14.05	56.60	25.91	6.76	15.51
	water capacity (cm/cm)		0.55	0.24	0.14	0.14	0.08	0.37	0.13	0.15	0.08
white oaks	subsection	1.00	1.00								
	geology	0.47									
	slope		0.38	2.43	1.72	2.49	2.30	2.74	1.80	2.28	2.25
yellow birch	subsection	1.00	1.00								
	geology	0.67	0.47								

In the Laurentian Mixed Forest, there were differences in mean values for predictions of ≥75% predicted probability of presence by species between GLO and FIA surveys even though subsection was the most important variable. Balsam fir, tamarack, white cedar, and yellow birch currently were present in sites with wetter soils (at least 10% greater than the GLO water capacity value), and the three pine species were present in drier sites compared to historical environmental conditions. Although slope was an important variable, slopes were not very steep and therefore most values did not change by more than 1% in slope (i.e., from 2.5 to 3.5% slope) between GLO and FIA models. The three pines and birch were restricted to sites with sandier soils; soils at current sites were greater by 6 to 19% in sand percentage than soils at historical sites. Conversely, spruces, tamarack, white cedar, and yellow birch were present at sites with soils that decreased by 6 to 25% in sand percentage compared to soils at historical sites. Birch, elms, balsam fir, maples, red oak, white oak, tamarack, white cedar, and yellow birch were present at sites with soils that increased by at least 10% of the GLO value for organic matter (up to a difference of 32 in soil organic matter percentage for tamarack) compared to organic matter at historical sites, whereas aspens, the three pines, and basswood were present at sites with soils that had less organic matter than in the past.

In the Eastern Broadleaf Forest, despite changes in predicted probabilities, important variables remained similar between GLO and FIA surveys based on subsection and topography, but also became less influential (Table 5). Birch, elms, cherries, and white oak shared only two of the five most influential variables in GLO and FIA models and American basswood and hickories shared one of the five most influential variables. For GLO surveys, subsection was the most common of the five most influential variables and it was present in 14 models (overall mean rank of 1.29), followed by elevation and % sand (one of the five most influential variables in 8 models each), geology, solar radiation, water capacity (7 models each), and % clay (6 models). For FIA surveys, subsection was one of the five most influential variables in 11 models (overall mean rank of 4.86), followed by slope (one of the five most influential variables in 9 models), solar radiation (8 models), wetness index (8 models), geology (7 models), and elevation and roughness index (6 models each).

**Table 5:** Two most influential predictor variables for GLO and FIA species distribution models by species and values of the predictor variables for predicted presence (probabilities ≥75%) and predicted absence (probabilities <75%) of the species in the Eastern Broadleaf Forest.

		GLO importance	FIA importance	GLO				FIA			
				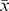	SD	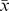	SD	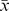	SD	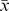	SD
				probabilities ≥75%		probabilities <75%		probabilities ≥75%		probabilities <75%	
American basswood	subsection	1.00									
	water capacity (cm/cm)	0.40		0.17	0.04	0.16	0.06	0.15	0.04	0.17	0.06
	pH		1.00	6.98	0.45	6.68	0.60	6.96	0.54	6.76	0.57
	elevation (m)		0.90	345.07	51.08	340.74	49.08	356.89	57.13	339.53	47.84
ashes	subsection	1.00									
	geology	0.74									
	slope (%)		1.00	3.05	1.65	6.16	6.55	3.24	2.67	5.64	6.09
	wetness		0.99	5.34	0.87	4.75	1.04	5.44	0.86	4.80	1.02
aspens	subsection	1.00									
	geology	0.63									
	elevation (m)		1.00	362.23	51.64	332.90	46.11	378.21	55.87	337.99	47.28
	solar radiation		0.90	5531.90	40.79	5497.99	71.06	5523.64	99.26	5507.14	59.06
birch	subsection	1.00	0.95								
	geology	0.51									
	slope (%)		1.00	6.65	9.08	4.65	4.20	12.79	11.59	4.39	4.06
cherries	subsection	1.00									
	pH	0.78		7.00	0.31	6.69	0.64	6.63	0.48	6.87	0.59
	elevation (m)		1.00	327.72	30.57	349.95	55.91	333.15	37.81	347.22	54.57
	geology		0.97								
eastern white pine	subsection	1.00									
	geology	0.82									
	position		1.00	−0.01	0.15	0.01	0.35	0.21	0.18	−0.01	0.34
	solar radiation		0.82	5570.13	11.28	5501.77	64.77	5512.13	23.52	5508.75	66.50
elms	subsection	1.00									
	clay (%)	0.50		22.28	8.06	18.87	9.30	23.31	7.72	18.78	9.18
	solar radiation		1.00	5520.55	36.93	5501.78	76.33	5476.45	91.07	5523.34	41.49
	slope (%)		0.78	3.65	2.42	5.90	6.64	7.92	8.09	3.76	3.16
hickories	subsection	1.00	1.00								
	sand (%)	0.70		30.32	10.93	48.41	26.52	23.69	17.55	45.39	23.90
	slope (%)		0.71	7.02	7.42	4.12	4.10	15.60	8.81	3.54	2.45
maples	subsection	1.00									
	geology	0.39									
	elevation (m)		1.00	340.96	52.72	342.90	48.76	347.11	58.41	341.80	48.75
	solar radiation		0.96	5519.50	37.24	5504.93	72.24	5507.16	76.09	5509.15	63.39
red oaks	wetness	1.00		4.57	0.93	5.24	0.99	4.31	1.12	5.07	0.96
	subsection	0.96									
	water capacity (cm/cm)		1.00	0.16	0.04	0.16	0.06	0.14	0.05	0.17	0.05
	organic matter (%)		0.94	1.39	4.16	4.72	11.08	1.50	5.12	3.65	9.50
spruces	subsection	1.00									
	solar radiation	0.72	0.94	5566.87	12.18	5497.71	64.94	5552.45	20.51	5490.90	68.31
	elevation (m)		1.00	416.85	19.90	327.94	40.14	391.65	30.93	321.94	41.23
tamarack	subsection	1.00									
	wetness	0.76		5.86	0.93	4.81	0.95	6.60	0.36	4.87	0.97
	organic matter (%)		1.00	13.38	19.26	1.60	3.31	38.51	14.70	1.40	1.63
	drainage		0.89								
walnuts	sand (%)	1.00		31.21	11.29	48.73	26.96	25.32	12.68	49.73	24.32
	subsection	0.91	1.00								
	geology		0.54								
white oaks	subsection	1.00									
	elevation (m)	0.50		347.10	42.52	339.33	53.88	366.17	50.91	337.11	48.10
	wetness		1.00	4.59	0.93	5.20	1.00	4.53	1.04	5.05	0.99
	solar radiation		0.98	5502.26	82.69	5513.23	49.70	5508.26	100.11	5509.09	54.05

There were more differences in influential variable values by species for the Eastern Broadleaf Forest than the Laurentian Mixed Forest. Most species became associated with steeper and more rugged slopes, where there was less solar radiation, perhaps due to relatively low human preference (i.e., harvest or land use restrictions) for these areas. Seven species (basswood, birch, elms, maples, red oaks, hickories, and walnuts) currently were restricted to sites that were steeper by at least 2% in slope percentage compared to historical environmental sites (e.g., hickories currently were associated with sites of about 16% slope that were greater by 9% in slope percentage than historical sites of about 7% slope). Most species were present in less sunny locations, particularly basswood, birch, elms, red oaks, white pine, hickories, spruces, and tamarack. Five species (basswood, aspens, hickories, walnuts, and white oak) were associated with sites that were at least 15 m greater in elevation than sites in the past and five species (birch, elms, red oak, spruces, and white pine) currently were present in sites that were at least 14 m lower in elevation than sites in the past; white pine was present in sites that decreased almost 100 m in elevation. Basswood, birch, elms, and hickories were present in drier sites (that received less water) and tamarack was present in wetter sites compared to historical sites. Aspens, red oak, white oak, and cherries were present at sites with soils that decreased by 5% in clay percentage compared to soils at historical sites. Elms, spruces, tamarack, cherries, hickories, and walnuts were present at sites with soils that decreased by 6% in sand percentage compared to soils at historical sites. Red oak, white oak and aspens were present at sites with soils that increased by at least 8% in clay percentage compared to soils at historical sites; white oak was present at sites with soils that increased from 33% to 52% clay percentage compared to soils at historical sites.

**Figure 2:**
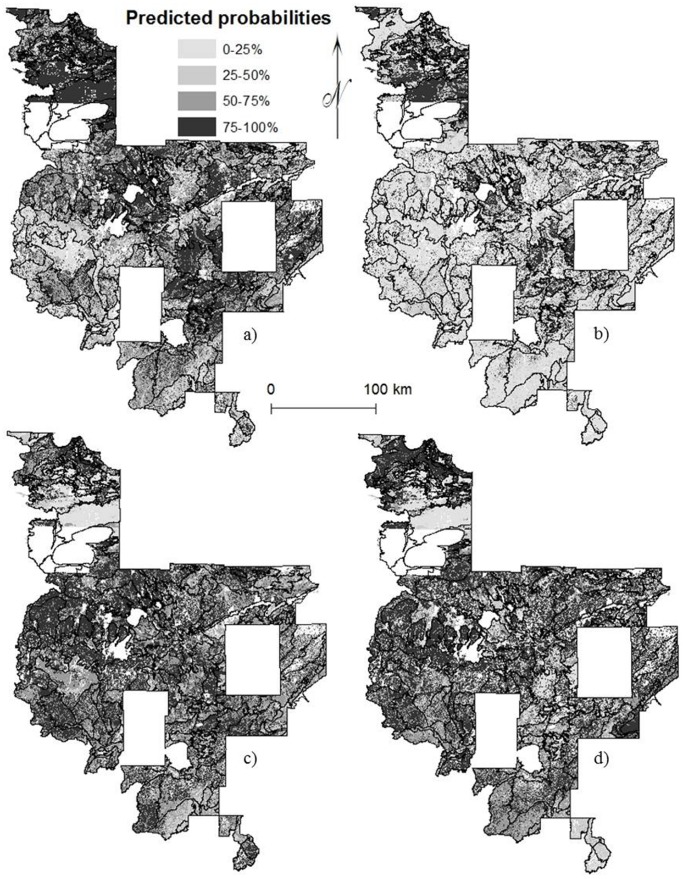
Predicted probabilities for (a) historical distribution of tamarack, (b) current distribution of tamarack, (c) historical distribution of aspens, and (d) current distribution of aspens in the Laurentian Mixed Forest. Tamarack range contracted whereas aspens maintained a similar range that was less uniform by ecological subsection and more varied within subsections.

**Figure 3:**
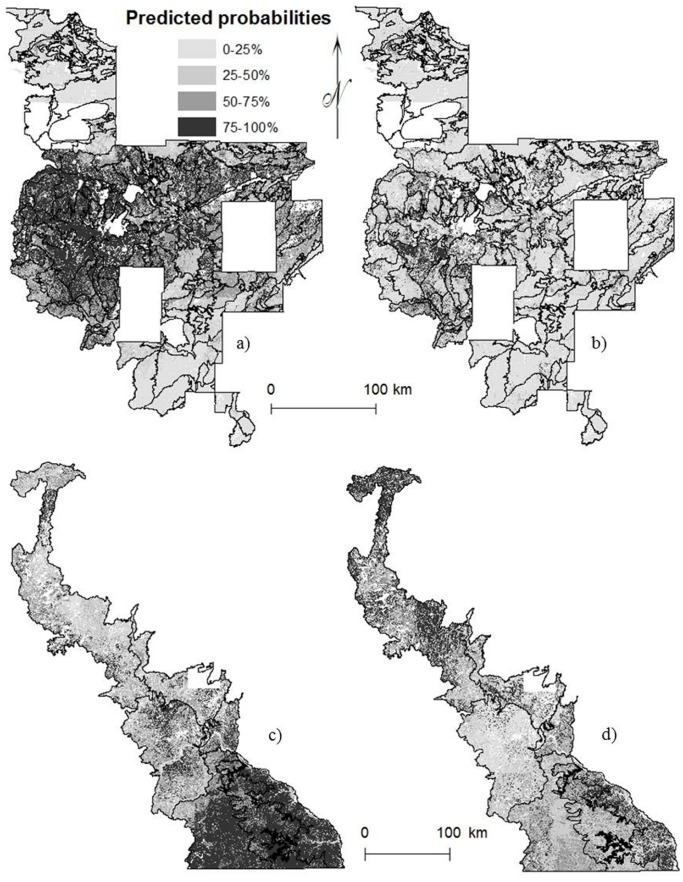
Predicted probabilities for (a) historical distribution of red pine and (b) current distribution of red pine in the Laurentian Mixed Forest and (c) historical distribution of white oaks and (d) current distribution of white oaks in the Eastern Broadleaf Forest. Red pine contracted in range whereas white oaks became less probable to occur and shifted northward.

Maps of species distributions displayed changes between historical and current forests; however the species distribution maps do not reflect density and indeed, appeared to reflect changes in influence of environmental gradients (Figures 2–3). Aspens, ashes, basswood, birch, elms, maples, and red oaks generally appeared to have a more uniform distribution in the Eastern Broadleaf Forest with less variation in predicted probability by subsection and maintained a similar range in the Laurentian Mixed Forest, but with finer scale variation in predicted probability within subsections. Tamarack and the three pines contracted in range. White oaks became less likely to occur, particularly in the southernmost portions of both provinces. Despite fairly similar historical and current abundance, spruce and fir probabilities declined at range edges and despite increasing cedar abundance, probabilities decreased resulting in a contraction of distribution.

### Winning and Losing Functional Traits

In the Laurentian Mixed Forest, coniferous species percent composition decreased from 65% in GLO surveys to 37% in FIA surveys for trees ≥12.7 cm and to 29% for trees of diameter <12.7 cm in FIA surveys. Pine percent composition decreased from 20% in GLO surveys to 9% in FIA surveys for trees ≥12.7 cm and 2.6% for trees <12.7 cm in FIA surveys. Oaks increased from 2.5% to 7%, probably increasing in the ideal conditions of open pine stands. Proportion of species with greater shade tolerance increased, as mean shade tolerance increased from 2.29 (±0.003) in GLO surveys to 2.50 (±0.006) in FIA surveys and remained about 2.42 (±0.010) for trees of diameter <12.7 cm in FIA surveys.

In the Eastern Broadleaf Forest, oak percent composition decreased from 54% in GLO surveys to 24% in FIA surveys for trees ≥12.7 cm and 6% for trees <12.7 cm in FIA surveys. Indeed, excluding the two subsections of the Hardwood Hills and Big Woods, which resemble current forests due to the presence of water bodies and other firebreaks, oak percent composition decreased from 86% (0.2% pine) in GLO surveys to 26% oak (10% pine) in FIA surveys for trees ≥12.7 cm and 8% oak (2.5% pine) for trees <12.7 cm in FIA surveys. Pine increased overall, probably due to successful plantations in two subsections. Proportion of species with greater shade tolerance increased, as mean shade tolerance increased from 2.62 (±0.003) in GLO surveys to 2.88 (±0.010) in FIA surveys and 3.01 (±0.023) for trees of diameter <12.7 cm in FIA surveys.

## Discussion

### Reassembly of Community Composition and Ecosystems

The Laurentian Mixed Forest of Minnesota transformed from forests where tamarack was most common, with subsections of spruce, birch, and jack pine dominance, to aspen-dominated forests with increased ashes, maples, balsam fir, and northern white cedar. Although we examined different extents of the Laurentian Mixed Forest, these results concurred with other research [23]–[25]. Composition has changed through replacement of gymnosperm species by angiosperm species and increased abundance of shade-tolerant species. Reassembly of communities from one type of early-successional forest ecosystem to another type of early-successional forest ecosystem may appear to be reassembly of communities rather than reassembly of forest ecosystem types because structure, function, and internal environmental feedback cycles remain relatively similar. Nevertheless, aspen are favored and stabilized by harvest in place of catastrophic stand-replacing fires, and aspen sustainability and production in turn promotes harvest and preference by the forest products industry. In addition, the trajectory of these ecosystems appears to include further increases in angiosperm species.

The Eastern Broadleaf Forest of Minnesota changed from open oak woodlands to oak forests that included ashes, American basswood, maples, boxelder, and red pine. Red pine increases probably were due to successful plantations. Continued loss of oaks and increases in shade-tolerant species appear to be the future trajectory of this region. Oak decline and replacement by fire-sensitive species is common throughout the eastern United States [4], [10], [26] and Minnesota is not an exception. Reassembly of forest ecosystems from species that are stabilized by and promote fire to species that compete well in the absence of fire and promote fire-extinguishing conditions is well-detailed [4], [27]–[28] even if not commonly documented across a landscape.

The General Land Office surveys may suffer from surveyor bias and it is possible that surveyor selection of preferred tree species influenced results [12]. We used 233,000 trees for the Laurentian Mixed Forest and 84,000 trees for the Eastern Broadleaf Forest, surveyed by many surveyors, and excluded unidentified pines and oaks from analysis. Small changes in composition of minor species may reflect only error and birch decreases may be misleading and due in part to possible preference for easily-blazed smooth-barked species by surveyors [12]. However, regional changes from 23% to 6% of composition (tamarack in the Laurentian Mixed Forest), 13% to 23% of composition (aspen in the Laurentian Mixed Forest), 38% to 14% of composition (white oaks in the Eastern Broadleaf Forest) and 0% to 7% (boxelder in the Eastern Broadleaf Forest) are large and match other sources of evidence in Minnesota and elsewhere [4], [10], [26]. We only compared changes in frequency, rather than accounting for volume using basal area, but diameters generally were greater historically than currently (B. Hanberry, unpublished data, University of Missouri). Unlike for species selection, there was systematic error for diameter due to surveyor instructions to select trees of moderate size [12].

### Environmental Gradients

Restrictions based on fine scale soils produced distributions that showed more variation within each subsection in the Laurentian Mixed Forest. Generally in the Laurentian Mixed Forest, as aspens increased in mesic sites away from wetlands, the three pine species (jack, red, white) became more restricted to sandy and drier sites, whereas tamarack, balsam fir, and white cedar became more constrained to wetlands. In the Eastern Broadleaf Forest, tree species lost differentiation among ecological subsections as the probability of presence became more even throughout all subsections. Furthermore, urbanization and other human land uses may be pushing all species to steeper slopes and less sunny locations. Dominant and declining oaks were present in sandier sites whereas declining tamarack was present in wetter sites, similarly to the Laurentian Mixed Forest. For both provinces, even though subsection became less influential in models of species presence, ashes and maples increased within ecological sites that may be similar to historical sites (that is, values for the influential variables in models were similar even if the influence of the variables was reduced), and therefore, other species such as aspen, basswood, and elms may be replacing oaks and pines at the drier spectrum of mesic sites.

Incidentally, researchers often implicate the larch sawfly (*Pristiphora erichsonii*) that invaded with Euro-American settlers as a reason for precipitous tamarack declines, just as the spruce budworm (*Choristoneura fumiferana*) can reduce balsam fir and spruce populations [29]–[31]. Tamarack decreased and became restricted to extremely wet conditions; bogs are now synonymous with tamarack. Unless larch sawfly is repelled by wet conditions, the pattern of tamarack decline does not seem to reflect decreases due to insects but rather systematic replacement by aspen in mesic sites. Balsam fir increased and therefore spruce budworm also does not seem to have been a problem. Instead of causing collapse of tree species, some researchers have suggested that insects such as the larch sawfly and spruce budworm, similarly to drought, generally cause overstory death that allows successful recruitment of advance regeneration into the canopy; even when sawfly larvae killed tamarack seedlings and saplings, a recruitment pulse from tamarack seeds occurred [29]–[32]. Death of the canopy reduced shade and created an opportunity for recruitment.

### Agreement between Disturbance Change and Reassembly in Species and Functional Groups

Extensive, selective, and intensive harvest instead of fire suppression may have been the major driver of reassembly in northern mixed forests, where historically stand-replacing fire regimes of 50 to 150 years re-initiated forests similar in composition and structure to previous forests, particularly early-successional tamarack forests. Fire-tolerant pines also were among the losing species, but pines were not as dominant as tamarack. In northern mixed forests, conifers decreased from 65 to 37% of composition and there were selective changes within the early-successional species group from the losing species of tamarack (decreased from about 23% to 6% of composition) to the winning species of aspen (increased from about 13% to 23% of composition). Harvest selection for pine and possibly tamarack followed by slash fires removed both seed sources and advance regeneration, allowing other pioneer species with light seeds or vegetative reproduction from roots to take the growing space vacated by conifers [6], [25]. Our findings support this compositional conversion and show that pine and tamarack were able to persist in sites at the extremes of soil moisture where they probably were less accessible to forestry operations. Harvest for economically valuable species prevented the opportunity for conifer regeneration and the eventual advent of sustainable forestry along with development of pulp products favored aspen over other pioneer species, such as birch.

Overall, proportion of species with greater shade tolerance increased, which indicates release of pressure from disturbance and suggests that current harvest does not remove as much tree biomass as past disturbances including fire. Although most shade-tolerant species were winning species, excluding dominant spruces, the primary winner was aspen, an early-successional species. Because of shifts among species in the same functional group due to assembly filters, functional groups of 1) gymnosperm or angiosperm class or 2) successional class based on shade tolerance could not completely explain winners and losers. However, in combination of the two functional groups, shade-tolerant angiosperm species increased and early-successional gymnosperms decreased.

In eastern broadleaf forests, oaks were the losing species while fire-sensitive species increased, similarly to other research [4], [10], [26], [33]–[34]. Because of historical dominance by fire-stabilized oak species, functional groups based on fire tolerance explained current winners and losers but further refinement by shade tolerance explains future trajectory of forests. Harvest potentially accelerated the effects of fire suppression, by allowing a dense pulse of oak recruitment to the canopy, which fostered ideal conditions for fire-sensitive species to establish in the understory in the absence of fire. As opposed to winning species colonizing empty growing space in the Laurentian Mixed Forest, winning species out-competed oaks for resources in the Eastern Broadleaf Forest. If fire suppression had not coincided with establishment of denser oak forests, then probably fire would have removed advanced regeneration of fire-sensitive species over time in most areas, but fire-sensitive species could have claimed sites that were less fire-prone at least in the short term. Silvicultural management has not been as successful in eastern broadleaf forests at maintaining fire-stabilized oaks against angiosperm shade-tolerant species as management in mixed forests has been at maintaining aspen in mixed forests against competition from gymnosperm and angiosperm shade-tolerant species. With fire suppression, canopy removal in oak forests that have an understory of mesic species simply allows fire-sensitive species to capture sites more quickly [9], [35]–[36].

### Future Trajectory

Both ecological theory and the weight of evidence, including our research, indicate that transition to increasingly shade-tolerant fire-sensitive species occurs in the absence of fire disturbance due to traits of fire-sensitive species that become more successful [4], [37]–[38]. Over the past century of effective fire suppression, eastern oak forest ecosystems, including in Minnesota, have been converting to forests of shade-tolerant species in ecological subsections that historically differentiated species by fire tolerance. In northern mixed forests, dense forests of early-successional species also have been converting to shade-tolerant species, but this trend in not as obvious as in eastern broadleaf forests because 1) fire-dependent pines were limited in extent compared to fire-dependent oaks in eastern broadleaf forests and 2) silvicultural practices are maintaining early-successional aspens against competition from shade-tolerant species. If fire suppression was more important than harvest in determining composition, then lack of disturbance would have resulted in continued succession of northern mixed forests to shade-tolerant mesic species, such as white spruce, black spruce, balsam fir, and northern white cedar along with ashes, maples, and basswood [37]–[38].

There were exceptions in the eastern United States, in which oaks were sustained where there was little evidence of fire history [39]. Xeric sites should resist mesophication for an extended period and careful silvicultural harvesting and discontinuous grazing also appear to maintain silvopastural oak fields and woodlands for extended periods, although perhaps not indefinitely without fire [40]–[41]. Open oak forests occur in Europe in densities ranging from scattered large trees in moderately-grazed fields to oak woodlands intensively maintained by coppicing and clearing of invading species [40]–[43]. Records of management by anthropogenic fire are present for European forests until generally the late 1700s (as late as mid-1800s), in addition to the use of slash and burn agriculture; however recent fire management may be limited to heathlands [44]–[45]. Over the past 100 to at least 400 years in Europe, it appears that dominant oaks (*Q. robur*; *Q. petraea*) have been converting to mesic, shade-tolerant species, such as ash (*F. excelsior*), elm (*U. glabra*) and beech (*Fagus sylvatica*), due to coppice abandonment and changes in grazing patterns [40], [43], [45]–[47].

In connection with changes in grazing patterns, deer densities clearly are too high in some northern mixed forests and eastern broadleaf forests, as conservation and re-stocking of deer allowed deer densities to rise to perhaps two to five times historical densities during the 1930s to 1960s in some locations [48]–[50]. In northern mixed forests of the Great Lakes, conifers such as northern white cedar (*Thuja occidentalis*) may be replaced by sugar maple (*Acer saccharum*) and in eastern broadleaf forests, oaks and maples are replaced by black cherry (*Prunus serotina*), American beech (*Fagus grandifolia*), and ash (*Fraxinus* spp.) or are not regenerating after harvest because of browsing pressure [48], [50]–[51]. Nevertheless, deer herbivory does not appear to explain the particular compositional shifts between historical and current forests in Minnesota. Heavy browsing favors fast-growing early-successional species [52] and may prevent tree establishment; however, reassembly of communities to a greater component of early-successional species and open states is not a regional trend. Indeed, proportion of species with greater shade tolerance increased for both regions, which indicates release of pressure from disturbance. There were overall decreases in evergreen species, which may be more vulnerable to heavy browsing due to year-round availability or slower growth; however, decreases did not represent evergreen species preferred by deer. Variations in plant response to browse make reports of palatability conflicting even within a region, nevertheless, white cedar at least appears to generally reported as palatable [50] and yet palatable white cedar increased in Minnesota. Pines and tamarack declined dramatically, yet probably are not preferred browse compared to aspen [44], which became the most dominant genus. Palatable oaks are declining but oak recruitment was successful for 5000 years with pressure from megaherbivores and furthermore, recruitment of other palatable species occurs with browsing pressure. Indeed, some amount of herbivory is beneficial to oaks until oak densities are too low to produce enough seeds. Herbivory reduces the amount of vegetation, which may help maintain open oak (or pine) woodlands and an herbaceous ground cover in the absence of fire or silvicultural disturbance to remove excessive biomass that blocks light [40], [53]–[54].

### Conclusions

Re-assembly of communities occurred in Minnesota’s Laurentian Mixed Forest and Eastern Broadleaf Forest after changes in disturbance placed new filters on tree species. Winning species in the Laurentian Mixed Forest were early-successional aspen and most shade-tolerant species, excluding spruces, and losing species generally were early-successional conifers. Due to pressure from harvest and other filters, winners and losers were not specific to functional groups. Winning species in the Eastern Broadleaf Forest were fire-sensitive and losing species were fire-stabilized oaks. Because of dominance by fire-stabilized oaks in eastern broadleaf forests, fire tolerance as a functional group and fire suppression as a change in disturbance provided a consistent and unifying process to explain conversion to fire-sensitive species.

The two different forest ecosystems may converge to successional forest ecosystems composed of increasingly shade-tolerant species as tree biomass accumulates due to reduced disturbance. Conversely, forestry is a disturbance that can slow transition to late-successional species, at least by maintaining aspen against competition from shade-tolerant species but not generally maintaining oaks against shade-tolerant species. Forestry appeared to the primary driver of aspen dominance in mixed forests, but without strong forestry pressure and a stand-replacing fire regime, mixed forests will transition to shade-tolerant species similarly to broadleaf forests. The current conversion from gymnosperm to angiosperm tree species may escalate in northern mixed forests, depending on whether shade-tolerant gymnosperms can compete against shade-tolerant hardwoods.
